# The association between parental involvement in developmental advance and mental health in Chinese preschoolers: a cross-sectional study

**DOI:** 10.3389/fpubh.2026.1677781

**Published:** 2026-01-29

**Authors:** Yan Li, Hongli Sun, Jiahua Liu, Jie Mi

**Affiliations:** 1 School of Economics and Management, Yan'an University, Yan’an, Shaanxi, China; 2 Xi'an Key Laboratory of Children's Health and Diseases, Xi'an Children's Hospital, Shaanxi Institute for Pediatric Diseases, National Regional Children's Medical Center (Northwest), Affiliated Children's Hospital of Xi'an Jiaotong University, Xi'an, Shaanxi, China; 3 Department of Clinical Laboratory, Xi'an Children's Hospital, National Regional Children's Medical Center (Northwest), Affiliated Children's Hospital of Xi'an Jiaotong University, Xi'an, Shaanxi, China; 4 Department of Pharmacy, Xi'an Children's Hospital, National Regional Children's Medical Center (Northwest), Affiliated Children's Hospital of Xi'an Jiaotong University, Xi'an, Shaanxi, China

**Keywords:** child mental health, non-linear relationship, parental involvement, StimQ scale, strengths and difficulties questionnaire

## Abstract

**Objective:**

This study examined the association between Parental Involvement in Developmental Advance (PIDA) and mental health in Chinese kindergarten children aged 3–6 years, specifically assessing how parental teaching activities relate to emotional and behavioral adjustments.

**Methods:**

A cross-sectional study in a western Chinese city involved 21,366 children from 189 kindergartens, selected via stratified cluster sampling. PIDA was assessed via the StimQ Scale, measuring parental involvement in teaching activities related to emergent literacy and math/spatial orientation. Children’s mental health was evaluated using the Strengths and Difficulties Questionnaire (SDQ), with outcomes operationalized as total difficulties scores and prosocial behavior scores.

**Results:**

Each unit increase in PIDA score was corresponded to a 2% lower risk of total difficulties (OR = 0.98; 95% CI: 0.97–0.99) and a 4% higher likelihood of prosocial behavior (OR = 1.04; 95% CI: 1.03–1.05). Non-linear relationships showed optimal benefits at PIDA scores of 12 for total difficulties and 11 for prosocial behavior, beyond which excessive involvement elevated the risk of total difficulties by 18% (OR = 1.18; 95% CI: 1.14–1.22) and reduced prosocial behavior by 2% (OR = 0.98; 95% CI: 0.96–0.99). Subgroup analyses further indicated stronger effects in specific demographics, such as families with lower socioeconomic status. Significant interactions were found between PIDA and parental education level and employment status.

**Conclusion:**

Balanced parental involvement enhances children’s mental health by reducing total difficulties and boosting prosocial behavior. Excessive involvement may have negative effects, highlighting the need for tailored interventions to optimize early childhood mental health.

## 1 Introduction

Child mental health, according to the World Health Organization, is not just as the absence of disorders, but as a comprehensive state of well-being that includes emotional, psychological, and social aspects (1). Emotional and behavioral difficulties in early childhood are robust predictors of later psychopathology, including anxiety, depression, and conduct disorders (2, 3). With the rising prevalence of mental disorders among children, early intervention during critical developmental windows—particularly ages 3–6, when neuroplasticity is high and foundational self-regulation skills emerge—is essential for mitigating long-term mental health burdens (4). Family environment plays a crucial role in children’s mental health such as parents’ mental health, their parenting styles, and the quality of their relationships with children (5). Parental involvement, particularly in teaching activities, is increasingly recognized as a key factor influencing not only academic readiness but also emotional and behavioral development in early childhood.

Parental Involvement in Developmental Advance (PIDA) refers to the multiple roles and various forms that parents play in the educational process of their children. In Chinese traditional culture, which emphasizes parents’ responsibility for their children’s education, parents typically show a high level of involvement (6). In the early childhood education stage, parents’ participation is usually manifested as their support and involvement in their children’s daily learning activities, such as reading together and game-based learning (7). Research indicates that parents’ educational involvement is not only related to children’s academic achievements but also to their well-being (8). By participating in their children’s education, parents furnish children with essential security and connection, which fosters their emotional and cognitive development (9). According to StimQ, PIDA mainly refers to parents’ involvement in teaching activities, which can be divided into two parts: PIDAA (Emergent Literacy), which includes activities like letter recognition and writing, and PIDAB (Math/Spatial Orientation), covering skills such as time recognition and basic arithmetic. Notably, the two PIDA components may differentially influence mental health. For instance, literacy-oriented activities (PIDAA) often involve verbal interaction and storytelling, which may support emotional understanding and self-expression. In contrast, math/spatial tasks (PIDAB) tend to be more structured and rule-based, potentially eliciting greater performance pressure, which may undermine autonomy and detract from emotional needs as overly directive or excessive involvement (9–14). The StimQ instrument has demonstrated a good reliability (full scale *α* = 0.88; PIDA subscale α = 0.68) in the original article (15).

The evidence regarding parental involvement’s impact extends beyond academic domains to encompass social and emotional development, though findings present notable contradictions. While some studies report positive associations with social competence, self-esteem, and self-regulation (16–19), others suggest that highly structured, directive involvement may potentially undermine autonomy development and intrinsic motivation (20, 21). Evidence regarding emotional regulation presents similar complexities: although warm, responsive involvement appears protective against anxiety and depression (22, 23), excessive academic focus within parent–child interactions may conversely elevate stress levels and diminish emotional well-being (24). Crucially, emerging evidence indicates that the quality of parental involvement demonstrates stronger associations with child adjustment than mere quantitative frequency (8), yet the mechanisms underlying this relationship remain inadequately explained, particularly regarding how structured teaching activities specifically affect mental health.

Despite growing interest, few large-scale studies have quantitatively examined the non-linear relationship between structured parental teaching activities and early childhood emotional and behavioral outcomes, particularly within the Chinese cultural context where parental involvement is highly emphasized. To overcome these limitations, we utilized a substantial population-based cohort consisting of kindergarten children from a city in China. We hypothesize that the association between PIDA and child mental health is non-linear, with moderate levels being beneficial, but excessive involvement predicting increased emotional and behavioral difficulties. The research uses a stratified cluster sampling method across different districts and includes a wide range of child and family characteristics, providing a comprehensive analysis of the association between PIDA and the mental health among children aged 3 to 6 years.

## 2 Methods

### 2.1 Ethical approval

Ethical approval was obtained from the Ethics Committee of the Affiliated Children’s Hospital of Xi’an Jiaotong University (Approval No. 20250225–21). Written informed consent was obtained from one parent (either the mother or father) per child, who completed the survey. Non-parental respondents, such as grandparents or foster parents, were excluded.

### 2.2 Study population

The target sample size of 28,000 was conservatively estimated based on an assumed odds ratio of 0.90. After accounting for a design effect of 2.0 (due to cluster sampling) and 20% anticipated attrition, the calculated sample size was increased from 20,000 to 28,000 to ensure adequate power under potential underestimation of clustering effects or higher attrition rates. This research was carried out in partnership with the Education Bureau and local schools in a city located in western China. Using stratified cluster sampling, the city’s 13 districts/counties were strata. Public kindergartens were chosen at random from each district based on an official list of kindergartens. Proportional allocation was used to calculate the number of participants from each district according to the ratio of children to the overall population. A total of 189 kindergartens were chosen as recruitment locations, and all children along with their parents from these institutions were invited to take part. Recruitment took place from February 28 to March 5, 2025. The research involved preschoolers between the ages of 3 and 6 who were attending junior, middle, and senior kindergarten classes. A total of 28,000 questionnaires were distributed, and 25,017 were returned, yielding a response rate of 89.3% (25,017/28,000). After removing responses that had outliers related to the age of the form fillers (517), non-parental respondents (2,408), and the age of the children (726), the final analysis comprised 21,366 participants.

### 2.3 Exposure

The exposure variable was PIDA, assessed using the StimQ Scale. StimQ links children’s healthy development to their home environment, influenced by elements like parental education, mental well-being, and financial circumstances (25, 26). StimQ is a dependable, efficient, and easy-to-use tool created for evaluating children younger than 6 years old (27). The StimQ comprises four subscales: Reading (READ), Parental Involvement in Developmental Advancement (PIDA), Parental Verbal Responsivity (PVR), and Availability of Learning Materials (ALM). This study focused solely on the PIDA subscale, which measures teaching activities and includes two subdimensions: PIDAA (Emergent Literacy) and PIDAB (Math and Spatial Orientation) (28). The PIDA subscale consists of 15 yes/no questions (7 for PIDAA, 8 for PIDAB), with 1 point awarded for a “yes” response and 0 for a “no” response. The overall PIDA score is calculated by adding the points from PIDAA and PIDAB, with higher scores indicating greater parental involvement in teaching activities. The Chinese version of the StimQ questionnaire, including its Parental Involvement in Developmental Advance (PIDA) subscale, was used in this study. The internal consistency reliability for this instrument in our sample was good, as indicated by a Cronbach’s alpha of 0.882.

### 2.4 Outcome

Our approach was grounded in established practices within the field, where the SDQ is a well-validated instrument for assessing mental health in children. As operationalized through the total difficulties and prosocial behavior scores, it is well-documented in both international (29–31) and specifically Chinese (32–35) contexts. In this study, mental health is used as an operational term to encompass the broad range of behavioral and emotional problems measured by the SDQ. It is not equated with a clinical diagnosis of a mental health disorder. This questionnaire includes 25 questions, with responses scored from 0 (“does not apply”) to 2 (“completely applies”). Questions 7, 11, 14, 21, and 25 are scored in reverse. The survey consists of five sections: emotional issues, behavioral problems, hyperactivity, difficulties with peers, and prosocial behavior. Based on the recommended cutoff values for the SDQ in China (36, 37), the total difficulties score is calculated based on four subscales: emotional issues, behavioral problems, hyperactivity, and difficulties with peers. The SDQ was treated as a dichotomous variable. A total difficulties score exceeding 14 suggests a potential risk for mental health. Meanwhile, a prosocial behavior score under 6 indicates a lack of prosocial behavior. It has shown high internal consistency and validity in evaluating mental health among Chinese children between the ages of 3 and 17 (37, 38). This research employs both the total difficulties score and the prosocial behavior score as dependent variables.

### 2.5 Covariates

In order to address possible confounding variables and clarify the connection between parental teaching activities and children’s mental health, the following covariates were gathered: parent respondent’s gender (male/female), parental age (continuous or categorical: < 40, ≥ 40 years), child age (continuous or categorical: < 4, ≥ 4 to < 5, ≥ 5 years), child gender (boys/girls), parental education level (≤ high school diploma, junior college, ≥ undergraduate degree), employment status (working/not working), annual family income (< ¥100,000, ≥ ¥100,000), smoking habits (yes/no), alcohol intake (yes/no), and marital status (married/living with partner, other). We selected these confounders on the basis of their associations with the outcomes of interest or a change in effect estimate of more than 10% or the *p*-value of the covariate’s regression coefficient for total difficulties/prosocial behavior is <0.1. Supplementary Table 1 showed the associations of each confounder with the outcomes of interest.

### 2.6 Statistical analysis

Continuous variables that followed a normal distribution were presented as mean ± standard deviation (SD), whereas categorical variables were shown as percentages. Baseline characteristics were stratified by total difficulties scores (> 14 vs. ≤ 14). The Kruskal-Wallis H test was utilized for comparing continuous variables, while Fisher’s exact test was employed for comparing categorical variables. Although participants were recruited from 189 kindergartens, the intraclass correlation coefficient (ICC) for both outcomes was negligible (ICC < 0.2%). Multilevel logistic regression models did not converge due to near-zero kindergarten-level variance, confirming that clustering effects were minimal. Therefore, standard logistic regression was used for all analyses (Supplementary Table 2).

Logistic regression analysis was performed to assess the connection between PIDA and mental health in children, with the findings presented as Odds Ratio (OR) and 95% Confidence Interval (CI). Multivariable logistic regression models were developed using a stepwise approach: the unadjusted model did not include any covariate adjustments, whereas the adjusted model took into consideration parent respondent’s gender, parental age, child age, child gender, education level, employment status, annual family income, smoking habits, alcohol intake, and marital status. Model fit was assessed using Brier scores, area under the ROC curve (AUC), classification accuracy, and Hosmer-Lemeshow tests. Overfitting was evaluated through 10-fold cross-validation, comparison of training versus testing set performance, Akaike Information Criterion (AIC), and variance inflation factors (VIF). Complete diagnostic results are presented in Supplementary Table 3. To explore potential non-linear relationships between PIDA and children’s mental health, a Generalized Additive Model (GAM) with smoothed curve fitting was employed.

Subgroup analyses were performed to examine potential effect modification by demographic and socioeconomic factors. To account for multiplicity due to multiple testing across 10 subgroup variables, a Bonferroni correction was applied. The family-wise error rate was controlled at *α* = 0.05, resulting in a corrected significance threshold of *p* < 0.005 (0.05/10) for interaction tests. We used multiple imputation, based on 5 replications and a chained equation approach method in the R MI procedure, to account for missing data (Supplementary Table 4).

All statistical analyses were performed using R (version 4.2.1) and EmpowerStats (X&Y Solutions, Inc., Boston, MA). A two-tailed *p* < 0.05 was considered statistically significant.

## 3 Results

### 3.1 Fundamental traits of the participants

Table 1 displays the initial characteristics of the participants in the study. The cohort included 21,366 children, with a mean age of 4.82 ± 0.89 years. Based on total difficulties score, children were divided into two groups: a no-difficulty group (score ≤14, 81.38%) and a difficulty group (score >14, 18.62%). The no-difficulty group had a significantly higher PIDA score than the difficulty group (11.42 ± 3.65 vs. 11.14 ± 4.20, *p* < 0.001). Groups differed significantly in all covariates except child age.

**Table 1:** General baseline characteristics of participants.

Characteristics	Mean + SD / N (%)	Total difficulties score ≤ 14 (*n* = 17,388)	Total difficulties score > 14 (*n* = 3,978)	*p*-value
Child age (years)	4.82 ± 0.89	4.82 ± 0.88	4.80 ± 0.90	0.256
Child gender				<0.001
Boys	11,062 (51.77%)	8,897 (51.17%)	2,165 (54.42%)	
Girls	10,304 (48.23%)	8,491 (48.83%)	1813 (45.58%)	
Parental age (years)	34.75 ± 4.56	34.81 ± 4.53	34.48 ± 4.68	<0.001
Parent respondent’s Gender				<0.001
Male	5,108 (23.91%)	3,961 (22.78%)	1,147 (28.83%)	
Female	16,258 (76.09%)	13,427 (77.22%)	2,831 (71.17%)	
Education level				<0.001
≤ High school diploma	9,834 (46.03%)	7,690 (44.23%)	2,144 (53.90%)	
Junior college	5,383 (25.19%)	4,447 (25.58%)	936 (23.53%)	
≥ Undergraduate degree	6,149 (28.78%)	5,251 (30.20%)	898 (22.57%)	
Employment status				<0.001
Working	15,642 (73.21%)	12,912 (74.26%)	2,730 (68.63%)	
Not working	5,724 (26.79%)	4,476 (25.74%)	1,248 (31.37%)	
Annual family income, in thousands, ¥				<0.001
< 100	17,238 (80.68%)	13,820 (79.48%)	3,418 (85.92%)	
≥ 100	4,128 (19.32%)	3,568 (20.52%)	560 (14.08%)	
Smoking status				<0.001
No	18,187 (85.12%)	14,952 (85.99%)	3,235 (81.32%)	
Yes	3,179 (14.88%)	2,436 (14.01%)	743 (18.68%)	
Marital status				<0.001
Married/Living with partner	20,761 (97.17%)	16,939 (97.42%)	3,822 (96.08%)	
Others	605 (2.83%)	449 (2.58%)	156 (3.92%)	
Alcohol intake status				<0.001
No	17,858 (83.58%)	14,718 (84.64%)	3,140 (78.93%)	
Yes	3,508 (16.42%)	2,670 (15.36%)	838 (21.07%)	
PIDA scale	11.37 ± 3.76	11.42 ± 3.65	11.14 ± 4.20	<0.001
PIDAA subscale	4.60 ± 2.33	4.60 ± 2.30	4.62 ± 2.48	
PIDAB subscale	6.76 ± 1.83	6.82 ± 1.75	6.52 ± 2.12	

Continuous variables are expressed as means ± standard deviations and categorical data are presented as number (percentage). The Kruskal-Wallis H test was utilized for comparing continuous variables, while Fisher’s exact test was employed for comparing categorical variables.

### 3.2 Association between PIDA and mental health in children

Table 2 presents the association between PIDA and mental health in children. Multivariable logistic regression analysis was employed to investigate this relationship. Both unadjusted and adjusted models demonstrated that higher PIDA scores were linked to a significantly reduced likelihood of mental health (all P for trend < 0.001). Specifically, for each one-unit increase in PIDA score, the risk of total difficulties decreased by 2% (OR = 0.98; 95% CI: 0.97–0.99), while the occurrence of prosocial behavior increased by 4% (OR = 1.04; 95% CI: 1.03–1.05). In addition to the primary models, standardized effect sizes including odds ratios per standard deviation, standardized beta coefficients, and risk differences were calculated to enhance the interpretability and comparability of the findings. For full results of these supplementary analyses, please refer to the Supplementary Table 5. Similarly, the PIDA dimensions—PIDAA and PIDAB—showed similar patterns. While PIDAA showed no significant association with a decreased risk of total difficulties (OR = 1.00; 95% CI: 0.99–1.02; *p* > 0.05), indicating a lack of clinical relevance, it was associated with a 4% increase in the occurrence of prosocial behavior (OR = 1.04; 95% CI: 1.03–1.05; *p* < 0.0001). In contrast, PIDAB was linked to an 8% decrease in the risk of total difficulties (OR = 0.92; 95% CI: 0.91–0.94) and an 11% increase in the occurrence of prosocial behavior (OR = 1.11; 95% CI: 1.10–1.13). Both logistic regression models demonstrated adequate fit with minimal evidence of overfitting (Supplementary Table 3). AUC values reflected limited discriminative ability, consistent with the observed small effect sizes (Supplementary Figure S1).

**Table 2:** Associations between PIDA and emotional/behavioral problems in preschool children.

Exposure	Non-adjusted model	Adjust model
Total difficulties score		
PIDA scale	0.98 (0.97, 0.99) < 0.0001	0.98 (0.97, 0.99) 0.0001
PIDAA subscale	1.00 (0.99, 1.02) 0.5583	1.00 (0.99, 1.02) 0.7445
PIDAB subscale	0.92 (0.90, 0.94) < 0.0001	0.92 (0.91, 0.94) < 0.0001
Prosocial behavior		
PIDA scale	1.05 (1.04, 1.06) < 0.0001	1.04 (1.03, 1.05) < 0.0001
PIDAA subscale	1.05 (1.04, 1.06) < 0.0001	1.04 (1.03, 1.05) < 0.0001
PIDAB subscale	1.13 (1.12, 1.15) < 0.0001	1.11 (1.10, 1.13) < 0.0001

Data are represented by OR (95% CI) *p* value. Non-adjusted model adjust for: None. Adjust model adjust for: parent respondent’s gender; parental age; child gender; child age; smoking status; alcohol intake status; education level; annual family income; employment status; marital status.

### 3.3 Non-linearity relationship between PIDA and mental health in children

**Figure 1:**
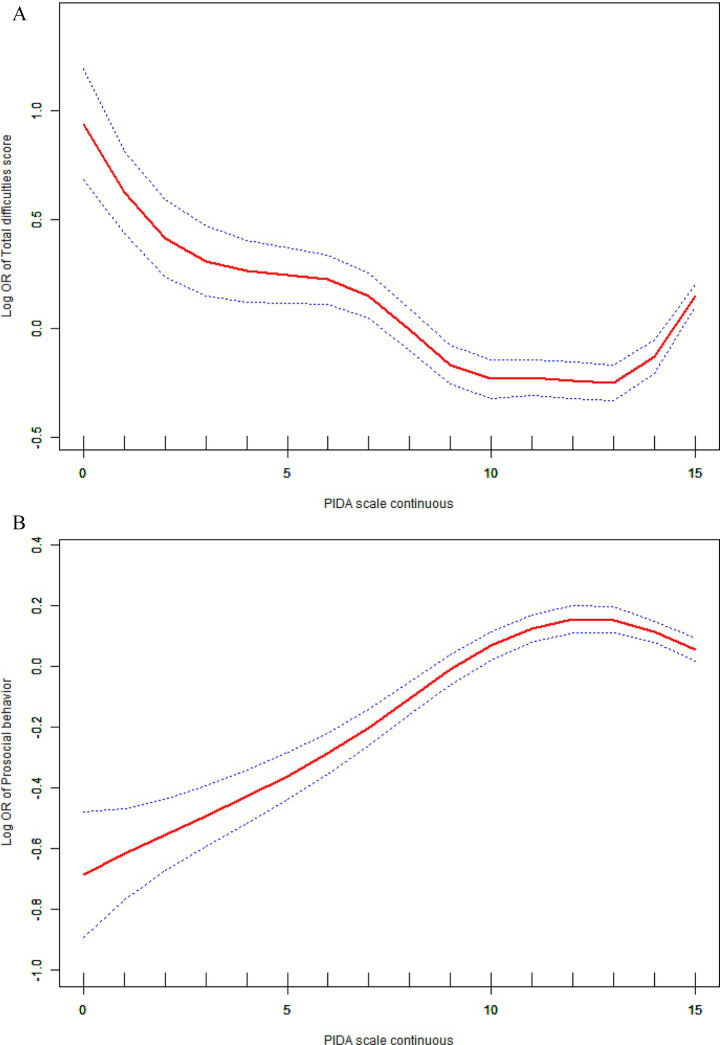
**(A)** Non-linearity relationship between PIDA and total difficulties in children. Adjusted for: parent respondent’s gender, age, education level, employment status, annual family income, smoking status, alcohol intake, and marital status, along with child age and gender. **(B)** Non-linearity relationship between PIDA and prosocial behavior in children. Adjusted for: parent respondent’s gender, age, education level, employment status, annual family income, smoking status, alcohol intake, and marital status, along with child age and gender.

The relationship between PIDA and children’s mental health was further explored through the use of smooth curve fitting, as depicted in Figure 1. Figure 1A shows the association with total difficulties, and Figure 1B with prosocial behavior. A non-linear relationship was observed after adjusting for covariates. Below a certain threshold, higher PIDA scores were associated with lower total difficulties and higher prosocial behavior.

The associations between PIDA and mental health were analyzed using two-piecewise linear regression and linear regression models, as presented in Table 3. A comparison of Akaike Information Criterion (AIC) between models confirmed the better fit of the piecewise linear model (Supplementary Table 6). The inflection points were identified at PIDA scores of 12 for total difficulties and 11 for prosocial behavior. For PIDA scores below 12, each one-unit rise in PIDA was corresponded to an 8% decrease in the risk of total difficulties (OR = 0.92, 95% CI: 0.90–0.93, *p* < 0.0001). Conversely, for PIDA scores ≥ 12, each one-unit rise was corresponded to an 18% increase in the risk of total difficulties (OR = 1.18, 95% CI: 1.14–1.22, *p* < 0.0001), showing a statistically significant reversal of the effect. Similarly, for PIDA scores below 11, each one-unit rise in PIDA increased the occurrence of prosocial behavior by 9% (OR = 1.09, 95% CI: 1.07–1.10, *p* < 0.0001). However, for PIDA scores ≥ 11, each one-unit increase reduced the occurrence of prosocial behavior by 2% (OR = 0.98, 95% CI: 0.96–0.99, *p* = 0.0091).

**Table 3:** Threshold effect analysis of PIDA scale on emotional/behavioral problems in preschool children by two-piecewise linear model.

Model	Total difficulties score	Prosocial behavior score
Fitting by the standard linear model	0.98 (0.97, 0.99) *	1.04 (1.03, 1.05) *
Fitting by the two-piecewise linear model		
Turning point (K)	12	11
< K segment effect 1	0.92 (0.90, 0.93) *	1.09 (1.07, 1.10) *
> K segment effect 2	1.18 (1.14, 1.22) *	0.98 (0.96, 0.99) *
Log likelihood ratio	<0.001	<0.001

Data are represented by OR (95% CI). * denotes significant correlations (*p* < 0.05). Adjust model adjust for: parent respondent’s gender; parental age; child gender; child age; smoking status; alcohol intake status; education level; annual family income; employment status; marital status.

### 3.4 Analysis of subgroups and testing for interactions

**Figure 2:**
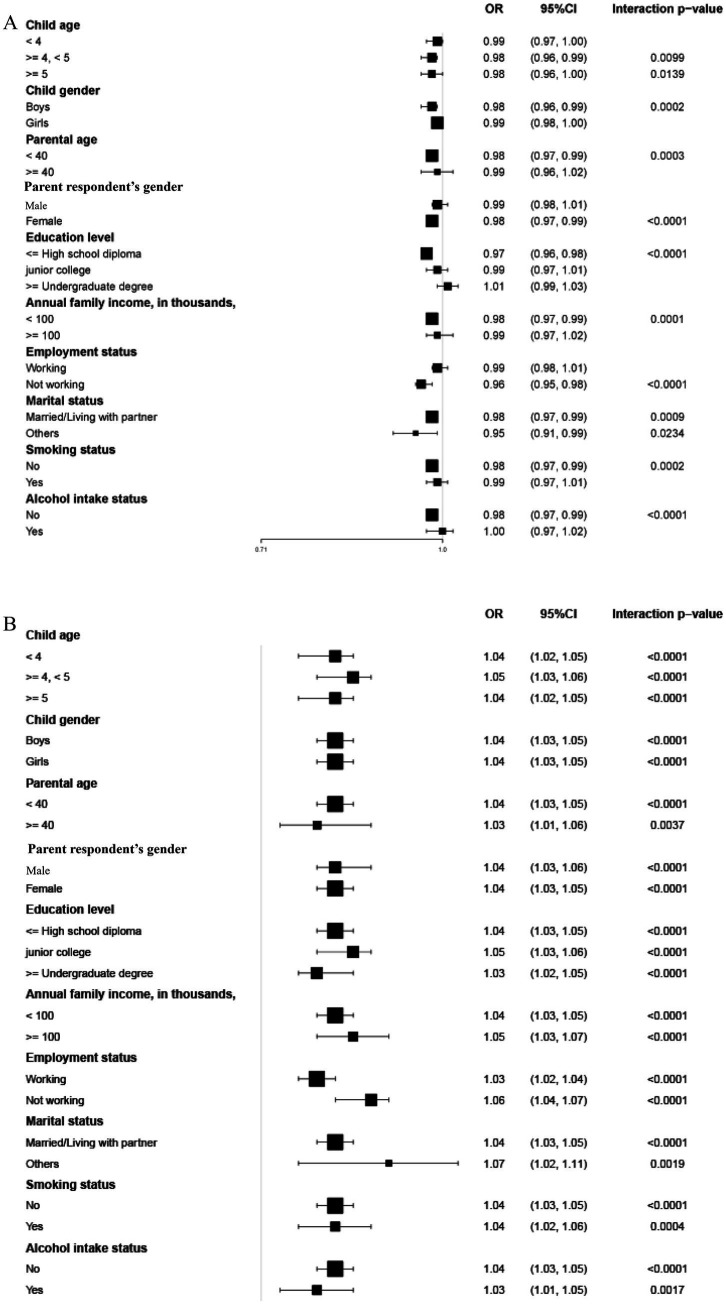
**(A)** Stratified analysis between PIDA and the risk of total difficulties in children. Adjusted for parent respondent’s gender, age, education level, employment status, annual family income, smoking status, alcohol intake, and marital status, along with child age and gender, except for the variable used in each stratified analysis. **(B)** Stratified analysis between PIDA and the occurrence of prosocial behavior in children. Adjusted for parent respondent’s gender, age, education level, employment status, annual family income, smoking status, alcohol intake, and marital status, along with child age and gender, except for the variable used in each stratified analysis.

Table 4 presents the stratified analysis of PIDA and child mental health by various demographic factors. This significant difference can be clearly observed in Figure 2. As shown in Figure 2A, the impact of PIDA on the risk of total difficulties was significant in children aged ≥ 4 years (≥ 4 to < 5 years: OR = 0.98, 95% CI: 0.96–0.99; ≥ 5 years: OR = 0.98, 95% CI: 0.96–1.00) but not in those aged < 4 years (OR = 0.99, 95% CI: 0.97–1.00). The association was significant in boys (OR = 0.98, 95% CI: 0.96–0.99, *p* = 0.0002) but not in girls (OR = 0.99, 95% CI: 0.98–1.00). Other significant associations were found for parents aged <40 years, mothers, parents with high school education or less, families with annual income <¥100,000, non-working parents, married/cohabiting parents, non-smokers, and non-drinkers. In contrast, the effect of PIDA on prosocial behavior was significant across all subgroups (all P for trend < 0.05), as shown in Figure 2B.

**Table 4:** Stratified analyses of the association between PIDA and emotional/behavioral problems in preschool children.

Characteristics	Total difficulties	P interaction	Prosocial behavior	P interaction
Child age		0.7163		0.4534
< 4	0.99 (0.97, 1.00)		1.04 (1.02, 1.05) *	
≥ 4 to 5	0.98 (0.96, 0.99) *		1.05 (1.03, 1.06) *	
≥ 5	0.98 (0.96, 1.00) *		1.04 (1.02, 1.05) *	
Child gender		0.1444		0.6034
Boys	0.98 (0.96, 0.99) *		1.04 (1.03, 1.05) *	
Girls	0.99 (0.98, 1.00)		1.04 (1.03, 1.05) *	
Parental age		0.7014		0.4706
< 40	0.98 (0.97, 0.99) *		1.04 (1.03, 1.05) *	
≥ 40	0.99 (0.96, 1.02)		1.03 (1.01, 1.06) *	
Parent respondent’s gender		0.1458		0.8634
Male	0.99 (0.98, 1.01)		1.04 (1.03, 1.06) *	
Female	0.98 (0.97, 0.99) *		1.04 (1.03, 1.05) *	
Education level		0.0016		0.5448
≤ High school diploma	0.97 (0.96, 0.98) *		1.04 (1.03, 1.05) *	
Junior college	0.99 (0.97, 1.01)		1.05 (1.03, 1.06) *	
≥ Undergraduate degree	1.01 (0.99, 1.03)		1.03 (1.02, 1.05) *	
Annual family income, in thousands, ¥		0.2842		0.2524
< 100	0.98 (0.97, 0.99) *		1.04 (1.03, 1.05) *	
≥ 100	0.99 (0.97, 1.02)		1.05 (1.03, 1.07) *	
Employment status		0.0005		0.0048
Working	0.99 (0.98, 1.01)		1.03 (1.02, 1.04) *	
Not working	0.96 (0.95, 0.98) *		1.06 (1.04, 1.07) *	
Marital status		0.1329		0.2202
Married/Living with partner	0.98 (0.97, 0.99) *		1.04 (1.03, 1.05) *	
Others	0.95 (0.91, 0.99) *		1.07 (1.02, 1.11) *	
Smoking status		0.3738		0.6090
No	0.98 (0.97, 0.99) *		1.04 (1.03, 1.05) *	
Yes	0.99 (0.97, 1.01)		1.04 (1.02, 1.06) *	
Alcohol intake status		0.1522		0.2455
No	0.98 (0.97, 0.99) *		1.04 (1.03, 1.05) *	
Yes	1.00 (0.97, 1.02)		1.03 (1.01, 1.05) *	

Data are represented by OR (95% CI). * denotes significant correlations (*p* < 0.05). Non-adjusted model adjust for: None. Adjust model adjust for: parent respondent’s gender; parental age; child gender; child age; smoking status; alcohol intake status; education level; annual family income; employment status; marital status. Stratification variables were not involved in the adjustment of the respective stratification analysis.

To control for multiple comparisons across the 10 tested interactions (Table 4), a Bonferroni correction was applied, setting the significance threshold at *p* < 0.005. After this adjustment, significant interactions were identified between PIDA and education level, as well as employment status for total difficulties. Employment status also modified the effect of PIDA on prosocial behavior. No other covariates showed significant interactions (all P interaction > 0.005).

## 4 Discussion

Mental health represents a major public health challenge, significantly adding to the worldwide disease burden (39). Issues such as anxiety, depression, and behavioral problems can significantly impede a child’s developmental trajectory, affecting their mental health and social interactions (40). Our study showed that greater parental involvement was linked to a lower risk of mental health in children, both before and after adjusting for various covariates. However, the non-linear findings suggest that over-involvement could lead to adverse outcomes, particularly if involvement becomes overly directive or stressful for children. Furthermore, parental education level and employment status interacted with parental involvement, affecting how parental teaching activities influence children’s total difficulties and prosocial behavior. These findings have significant implications for early childhood education and mental health interventions.

The analysis revealed that a higher PIDA score was linked to a significantly decreased risk of total difficulties and an increased occurrence of prosocial behavior in children. Specifically, for each one-unit rise in PIDA score, the risk of total difficulties decreased by 2%, while the occurrence of prosocial behavior increased by 4%. This finding is consistent with prior research, demonstrating that family involvement in early childhood education influences the social–emotional development and behaviors problem of 3-year-old children (16). Parental involvement in education can improve preschoolers’ social skills, particularly when parents and children communicate and interact effectively (41). Parents who participate in family activities, like playing games and studying together, can boost their children’ self-confidence (42). Additionally, children with involved parents exhibit better social functioning and fewer behavior issues (43). According to SDT (Self-Determination Theory), children’s need for relatedness and support is fundamental to psychological development. Positive parental involvement strengthens emotional bonds and promotes a sense of security and belonging, which are vital for mental health and adaptive social engagement (44, 45). Notably, the PIDA subscale focusing on PIDAB showed a stronger association with reduced total difficulties and increased prosocial behavior compared to PIDAA, suggesting that structured activities involving numerical and spatial skills may have a unique impact on mental health. Mathematics often poses particular difficulty and pressure for students compared to other subjects such as literacy and language skills (9). In this context, supportive parental involvement that provides autonomy can enhance children’s intrinsic motivation, thereby fostering self-efficacy and emotional well-being (13).

We applied smooth curve fitting to characterize how PIDA relates to children’s mental health. Notably, the relationships between PIDA and both total difficulties and prosocial behavior are non-linear. A higher PIDA score does not always indicate better outcomes. Below a PIDA score of 12 for total difficulties and 11 for prosocial behavior, increased parental involvement was associated with improved mental health. However, beyond these thresholds, further parental involvement increases the risk of total difficulties and decreases the likelihood of prosocial behavior. It is critical to emphasize that while the overall trend suggests benefits of parental involvement, the inversion of the effect after these thresholds is a key finding. This indicates an “optimal zone” for parental engagement, where moderate, high-quality involvement is most beneficial, while excessive involvement may diminish returns or even cause harm. The reversal of beneficial effects may reflect heightened psychological pressure and reduced autonomy support, particularly in cultures emphasizing academic achievement, where excessive involvement can become intrusive and stress-inducing. In China, where Confucian teachings are central, education is regarded as a moral pursuit (46). As a result, qualities such as diligence, perseverance, concentration, and hard work are deemed essential (6). In the context of education, Chinese parents often employ more instructions and commands when helping their children during their preschool years (47). Parental involvement often comes with increased control and reduced support for autonomy. As a result, parental involvement might not enhance children’s views of their own abilities and emotional health, especially when parents supply extra learning resources and push homework beyond its usual limits (6). In fact, parental involvement in children’s education can interfere with their psychological and emotional development (48). This finding underscores the importance of balanced parental involvement, where quality and appropriateness of engagement outweigh sheer quantity, as supported by prior studies (8). Our findings provide empirical support for the phenomenon often termed the “too much involvement effect” or parental overcontrol, demonstrating that involvement beyond an optimal level may produce counterproductive outcomes due to increased pressure, diminished autonomy, or unrealistic expectations (49, 50). This pattern aligns with clinical observations linking overcontrol to various early childhood psychiatric presentations, including anxiety, obsessive-compulsive and internalizing symptoms (51, 52), as well as later-emerging conditions such as anorexia nervosa and certain personality pathologies (53). Therefore, parents should avoid excessive involvement in their children’s teaching activities, as this may produce negative effects.

Subgroup analyses revealed variations in the impact of PIDA across demographic and socioeconomic factors. Specifically, PIDA was linked to a lower risk of total difficulties, especially in children aged 4 years and older, boys, parents under 40, mother, individuals who have completed high school or lower, families earning less than ¥100,000 annually, individuals not currently employed, non-smokers, and non-drinkers. These results indicate that PIDA could be especially advantageous in contexts where resources or opportunities for alternative support are limited. For instance, in lower-income families or among non-working parents, direct parental involvement may compensate for external resource constraints, providing critical emotional and cognitive support (54). Meanwhile, PIDA was linked to a higher frequency of prosocial behavior across all subgroups. Additionally, our analyses identified multiple statistically significant interactions in the relationships between PIDA and mental health in children. In terms of total difficulties, parental involvement showed significant interactions with parental education level and employment status. For prosocial behaviors, parental involvement interacted significantly with parental employment status. Parents’ education level is a socio-economic factor that influences children’s mental health (55–58). Numerous studies indicate parents’ education level predict their children’s mental health (59–61). Limited job opportunities stemming from low education levels can lead to mental health problems, creating a reciprocal relationship (62). Research shows that unstable or low-quality jobs are linked to poorer mental health in adults (63–65). Poor mental health among adults can have an adverse effect on the mental health of children (66). These interactions suggest that the influence of parental involvement is moderated by family resources and time availability. The protective effect of PIDA against total difficulties was more pronounced among parents with lower education levels and non-working parents. This pattern may be attributed to parental involvement compensating for limited access to alternative educational resources in these families, while also reflecting the greater availability of child-centered interactions that support the development of social–emotional skills (55, 56, 67). These significant interactions indicate that the effectiveness of parental involvement is context-dependent. The statistically significant but modest effect sizes observed in the forest plots are consistent with the complex, multifactorial nature of child mental health. Rather than diminishing the importance of parental involvement, these findings reinforce two key conclusions of our study. First, the non-linear threshold effects demonstrate that benefits are not simply dose-dependent, and excessive involvement can be detrimental. Second, the significant subgroup interactions indicate that effects are context-dependent, with meaningful variations across families. Thus, the primary implication is not to promote more involvement universally, but to support balanced and responsive parenting strategies tailored to family circumstances.

### 4.1 Strengths and limitations

The robustness of this study is enhanced by its substantial sample size and the application of validated instruments to evaluate both parental involvement and children’s mental health. The study employs a stratified cluster sampling technique to ensure that the sample accurately represents the population of kindergarten children in a Western city in China, thereby improving the applicability of the results. Additionally, the research takes into account various confounding factors such as parent respondent’s gender, age, education level, employment status, annual family income, smoking habits, alcohol intake, and marital status, as well as child age and gender. This approach provides a deeper insight into the elements that affect children’s mental health.

Nonetheless, it is important to recognize certain limitations. First, the cross-sectional design precludes causal inference. Longitudinal studies are necessary to clarify the direction of these connections. Second, our study relied on parental self-reported data, which may be subject to potential cultural biases in reporting, such as social desirability bias. This could lead parents to underreport or overreport certain behaviors, possibly affecting the validity of the findings. Third, the cultural specificity of the sample limits generalizability beyond China, as findings may not apply to other contexts. Finally, while the research took into account different demographic and family-related factors, it did not evaluate other possible confounding variables like neighborhood conditions or social support, and the absence of teacher reports or clinical assessments may constrain the robustness of emotional and behavioral evaluations.

## 5 Conclusion

This study demonstrates that parental involvement in developmental advance, as measured by the PIDA subscale, is strongly linked to better mental health in Chinese kindergarten children aged 3–6 years. Specifically, more parental involvement was associated with a significantly decreased risk of total difficulties and an increased occurrence of prosocial behavior in children. The non-linear relationship we observed serves as a key cautionary element, indicating that the benefits are not indefinite and may diminish or reverse beyond an optimal level of involvement. These findings highlight the importance of high-quality, balanced parental involvement in enhancing children’s mental health. Given the complexity of developmental influences, future research should prioritize longitudinal designs incorporating multilevel models to better disentangle within- and between-family dynamics, confirm the direction of causality, and clarify the mechanisms underlying these associations across diverse cultural and developmental contexts.

## Data Availability

The data analyzed in this study is subject to the following licenses/restrictions: this data set is still being used for analysis. Please contact the corresponding author regarding access to the full dataset. Requests to access these datasets should be directed to Jie Mi, mijie666@163.com.
