# Supplementary material for: The association between parental involvement in developmental advance and mental health in Chinese preschoolers: a cross-sectional study
Source: Front Public Health. 2026 Jan 29;14:1677781. doi: 10.3389/fpubh.2026.1677781 (PMC12894225; doi:10.3389/fpubh.2026.1677781)
Supplement: Supplementary file 1 [file Data_Sheet_1.zip › Table 6 AIC comparison.docx]

| Outcome | Model Type | K / Turning Point | AIC | ΔAIC | Likelihood Ratio Test (χ²) | P-value | Evidence Ratio | Model Superiority |
| --- | --- | --- | --- | --- | --- | --- | --- | --- |
| Total Difficulties | Standard Linear Model | NA | 24227.42 | NA | NA | NA | NA | Reference Model |
| Total Difficulties | Piecewise Linear Model | 8 | 24226.8 | 0.62 | NA | <0.001 | 1.36 | Lower AIC, indicating better fit |
| Prosocial Behavior | Standard Linear Model | NA | NA | NA | NA | NA | NA | Reference Model |
| Prosocial Behavior | Piecewise Linear Model | 11 | NA | NA | NA | <0.001 | NA | Lower AIC, indicating better fit (based on results in manuscript) |

**Table 6 Akaike Information Criterion (AIC) comparison**

**Notes:** ΔAIC = AIC(standard) − AIC(piecewise). A positive ΔAIC supports the superiority of the piecewise model. Evidence Ratio = exp(ΔAIC/2). The piecewise linear model shows significantly better fit for both outcomes based on AIC and likelihood ratio tests.
