# Supplementary material for: The association between parental involvement in developmental advance and mental health in Chinese preschoolers: a cross-sectional study
Source: Front Public Health. 2026 Jan 29;14:1677781. doi: 10.3389/fpubh.2026.1677781 (PMC12894225; doi:10.3389/fpubh.2026.1677781)
Supplement: Supplementary file 1 [file Data_Sheet_1.zip › Table 1 Covariate Check and Screening.docx]

**Table 1 Covariate Check and Screening**

Table S1 VIF Collinearity Screening

|  | Step 1 |
| --- | --- |
| PIDA | 1.1 |
| Parental age | 1 |
| Child age | 1.1 |
| Parent respondent’s gender | 2.4 |
| Child gender | 1 |
| Education level | 1.3 |
| Employment status | 1.3 |
| Marital status | 1 |
| Smoking status | 2.3 |
| Alcohol intake status | 1.8 |
| House hold in come each year | 1.1 |

Table S2 Examine the relationship between each covariate and Total difficulties individually.

| Covariates | N | term | beta | Se. | Exp (beta) | 95%CI Low | 95%CI Upp | P. value |
| --- | --- | --- | --- | --- | --- | --- | --- | --- |
| Education level | 21366 | Factor (Education level)2 | -0.4311 | 0.0411 | 0.6498 | 0.5995 | 0.7043 | <0.0001 |
|  |  | Factor (Education level)3 | -0.6396 | 0.0481 | 0.5275 | 0.4800 | 0.5797 | <0.0001 |
| Employment status | 21366 | Factor (Employment status)1 | -0.3854 | 0.0407 | 0.6802 | 0.6281 | 0.7366 | <0.0001 |
| Marital status | 21366 | Factor (Marital status1)1 | -0.3573 | 0.0956 | 0.6996 | 0.5800 | 0.8438 | 0.0002 |
| Smoking status | 21366 | Factor (Smoking status)1 | 0.1451 | 0.0675 | 1.1561 | 1.0128 | 1.3197 | 0.0317 |
| Alcohol intake status | 21366 | Factor (Alcohol intake status)1 | 0.2598 | 0.0568 | 1.2967 | 1.1600 | 1.4494 | <0.0001 |
| Household income each year | 21366 | Factor (House hold in come each year)2 | -0.3856 | 0.0387 | 0.6800 | 0.6303 | 0.7337 | <0.0001 |
|  |  | Factor (House hold in come each year)3 | -0.6550 | 0.0526 | 0.5194 | 0.4685 | 0.5759 | <0.0001 |

Table S3 Introduce covariates into the basic model and remove covariates from the full model, observe the change in the regression coefficient of PIDA.

|  |  | Basic Model | Full Model |  |
| --- | --- | --- | --- | --- |
| Covariate | +/- term | PIDA | PIDA | Selected |
|  | Starting Coefficient | -0.0235 | -0.0186 |  |
| Education level | Factor (Education level) | -0.0206 * | -0.0191 | Yes |
| Employment status | Factor (Employment status) | -0.0200 * | -0.0196 | Yes |
| Marital status | Factor (Marital status) | -0.0232 | -0.0187 |  |
| Smoking status | Factor (Smoking status) | -0.0234 | -0.0186 |  |
| Alcohol intake status | Factor (Alcohol intake status) | -0.0231 | -0.0191 |  |
| Household income each year | Factor (Household income each year) | -0.0222 | -0.0186 |  |

* Indicates a change exceeding 10% compared to the starting coefficient.

Table S4 Examine the relationship between each covariate and Prosocial behavior individually.

| Covariates | N | term | beta | Se. | Exp (beta) | 95%CI Low | 95%CI Upp | P.value |
| --- | --- | --- | --- | --- | --- | --- | --- | --- |
| Education level | 21366 | Factor (Education level)2 | 0.1591 | 0.0338 | 1.1724 | 1.0973 | 1.2527 | <0.0001 |
|  |  | Factor (Education level)3 | 0.2384 | 0.0375 | 1.2692 | 1.1793 | 1.3660 | <0.0001 |
| Employment status | 21366 | Factor (Employment status)1 | 0.1322 | 0.0324 | 1.1414 | 1.0711 | 1.2162 | <0.0001 |
| Marital status | 21366 | Factor (Marital status)1 | -0.1627 | 0.0837 | 0.8498 | 0.7213 | 1.0013 | 0.0519 |
| Smoking status | 21366 | Factor (Smoking status)1 | -0.0650 | 0.0561 | 0.9370 | 0.8395 | 1.0459 | 0.2462 |
| Alcohol intake status | 21366 | Factor (Alcohol intake status)1 | -0.1778 | 0.0471 | 0.8371 | 0.7633 | 0.9180 | 0.0002 |
| Household income each year | 21366 | Factor (Household income each year)2 | 0.1075 | 0.0307 | 1.1134 | 1.0483 | 1.1826 | 0.0005 |
|  |  | Factor (Household income each year)3 | 0.3098 | 0.0385 | 1.3631 | 1.2640 | 1.4700 | <0.0001 |

Table S5 Introduce covariates into the basic model and remove covariates from the full model, observe the change in the regression coefficient of PIDA

|  |  | Basic Model | Full Model |  |
| --- | --- | --- | --- | --- |
| Covariate | +/- term | PIDA | PIDA | Selected |
|  | Starting Coefficient | 0.0415 | 0.0403 |  |
| Education level | Factor (Education level) | 0.0407 | 0.0404 |  |
| Employment status | Factor (Employment status) | 0.0406 | 0.0403 |  |
| Marital status | Factor (Marital status) | 0.0417 | 0.0401 |  |
| Smoking status | Factor (Smoking status) | 0.0415 | 0.0403 |  |
| Alcohol intake status | Factor (Alcohol intake status) | 0.0413 | 0.0405 |  |
| Household income each year | Factor (Household income each year) | 0.0409 | 0.0404 |  |

* Indicates a change exceeding 10% compared to the starting coefficient.

Table S6 Selected Covariates

| Y | X | Selected Covariates |
| --- | --- | --- |
| Total difficulties | PIDA | education level; employment status; marital status; smoking status; alcohol intake status; household income each year |
| Prosocial behavior | PIDA | education level; employment status; marital status; alcohol intake status; household income each year |

Note:

Selection criteria: The influence on the regression coefficient of PIDA when introducing the covariate into the basic model OR removing it from the full model is >10%, OR the P-value of the covariate's regression coefficient for total difficulties/ prosocial behavior is <0.1.

For the model of each covariate on total difficulties/prosocial behavior, the basic model, and the full model, the following variables were always adjusted for: Parental age; Child age; Parent respondent’s gender; Child gender.
