# Supplementary material for: The association between parental involvement in developmental advance and mental health in Chinese preschoolers: a cross-sectional study
Source: Front Public Health. 2026 Jan 29;14:1677781. doi: 10.3389/fpubh.2026.1677781 (PMC12894225; doi:10.3389/fpubh.2026.1677781)
Supplement: Supplementary file 1 [file Data_Sheet_1.zip › Table 2 Comprehensive results of clustering analyses..docx]

**Table 2: Comprehensive results of clustering analyses**

| **Analysis Item** | **Result** | **Interpretation** |
| --- | --- | --- |
| **Sample Information** | | |
| Total Sample Size | 21,366 | Total number of study participants. |
| Number of Kindergartens | 189 | Number of kindergartens included in the stratified sampling. |
| Average Sample Size per Kindergarten | 113 | Average number of participants per kindergarten. |
| **Kindergarten-Level Variance (ICC)** | | |
| Total Difficulties - ICC Value | 0.18% (ICC = 0.0018) | Intraclass Correlation Coefficient, measuring between-kindergarten variance. |
| Prosocial Behavior - ICC Value | 0% (ICC = 0) | Intraclass Correlation Coefficient, measuring between-kindergarten variance. |
| ICC Interpretation | Between-kindergarten variance < 5%, indicating minimal clustering effect. | An ICC < 5% suggests low necessity for multilevel modeling. |
| **Standard Logistic Regression Results** | | |
| Total Difficulties - PIDA Effect (OR) | 0.982 | Odds Ratio for total difficulties associated with a one-unit increase in PIDA score. |
| Total Difficulties - 95% CI | 0.973 - 0.991 | Precision of the effect estimate. |
| Total Difficulties - P-value | <0.001 | Statistical significance. |
| Prosocial Behavior - PIDA Effect (OR) | 1.038 | Odds Ratio for high prosocial behavior associated with a one-unit increase in PIDA. |
| Prosocial Behavior - 95% CI | 1.030 - 1.046 | Precision of the effect estimate. |
| Prosocial Behavior - P-value | <0.001 | Statistical significance. |
| **Multilevel Model Diagnostics** | | |
| Model Convergence Status | Attempted to fit but variance approached zero. | Multilevel logistic regression failed to converge stably. |
| Singular Model Check | Yes (model singular, variance component ≈ 0). | Random effect variance near zero, model simplifies to standard logistic regression. |
| Kindergarten Variance Component | Negligible (close to zero). | Between-kindergarten variation is negligible. |
