# Supplementary material for: The association between parental involvement in developmental advance and mental health in Chinese preschoolers: a cross-sectional study
Source: Front Public Health. 2026 Jan 29;14:1677781. doi: 10.3389/fpubh.2026.1677781 (PMC12894225; doi:10.3389/fpubh.2026.1677781)
Supplement: Supplementary file 1 [file Data_Sheet_1.zip › Table 3 Comprehensive model diagnostics.docx]

**Table 3 Comprehensive model diagnostics**

| **Diagnostic Measure** | **Value** | **Interpretation** | |
| --- | --- | --- | --- |
| Sample size | 21,366 | Total number of participants | |
| Prevalence of total difficulties | 18.60% | Percentage of children with total difficulties score > 14 | |
| Prevalence of high prosocial behavior | 50.60% | Percentage of children with prosocial behavior score < 6 | |
| Mean PIDA score (SD) | 11.37 (3.76) | Mean and standard deviation of Parental Involvement in Developmental Advance score | |
| PIDA range | 0 - 15 | Minimum and maximum observed PIDA scores | |
| **Overfitting Assessment** | | |  |
| 10-fold cross-validation AUC (mean ± SD) | 0.521 ± 0.008 (Total difficulties) \| 0.566 ± 0.007 (Prosocial behavior) | Stability of model performance across validation folds |  |
| Training vs testing AUC difference | -0.024 (Total difficulties) \| -0.015 (Prosocial behavior) | Differences < 0.05 indicate low overfitting risk |  |
| AIC difference (full vs reduced model) | 20517 (full) vs 20520 (intercept-only) | Small difference suggests minimal overfitting |  |
