# Supplementary material for: The association between parental involvement in developmental advance and mental health in Chinese preschoolers: a cross-sectional study
Source: Front Public Health. 2026 Jan 29;14:1677781. doi: 10.3389/fpubh.2026.1677781 (PMC12894225; doi:10.3389/fpubh.2026.1677781)
Supplement: Supplementary file 1 [file Data_Sheet_1.zip › Table 4 Multiple Imputation.docx]

**Table 4 Multiple Imputation**

Table S1 Distribution of missing variables.

| Characteristic | Missing number |
| --- | --- |
| Parental age | 517 |
| Child age | 726 |
| Non-parental respondents | 2408 |

Table S2 Five sets of regression data after interpolation for missing data on parental age, child age and non-parental respondents

| Exposure | Non-adjusted | Non-adjusted | Non-adjusted | Non-adjusted | Non-adjusted | Adjust model | Adjust model | Adjust model | Adjust model | Adjust model |
| --- | --- | --- | --- | --- | --- | --- | --- | --- | --- | --- |
| Total difficulties | 0.98 (0.97, 0.99) <0.0001 | 0.98 (0.97, 0.99) <0.0001 | 0.98 (0.97, 0.99) <0.0001 | 0.98 (0.97, 0.99) <0.0001 | 0.98 (0.97, 0.99) <0.0001 | 0.98 (0.97, 0.99) <0.0001 | 0.98 (0.97, 0.99) <0.0001 | 0.98 (0.97, 0.99) <0.0001 | 0.98 (0.97, 0.99) <0.0001 | 0.98 (0.97, 0.99) <0.0001 |
| Prosocial behavior | 1.05 (1.04, 1.05) <0.0001 | 1.05 (1.04, 1.05) <0.0001 | 1.05 (1.04, 1.05) <0.0001 | 1.05 (1.04, 1.05) <0.0001 | 1.05 (1.04, 1.05) <0.0001 | 1.04 (1.03, 1.05) <0.0001 | 1.04 (1.03, 1.05) <0.0001 | 1.04 (1.03, 1.05) <0.0001 | 1.04 (1.03, 1.05) <0.0001 | 1.04 (1.03, 1.05) <0.0001 |

Note: OR (95%CI) P value
Non-adjusted model adjust for: None
Adjust model adjust for: parent respondent’s gender; parental age; child gender; child age; smoking status; alcohol intake status; education level; annual family income; employment status; marital status.

Table S3 Association of PIDA and total difficulties and prosocial behavior after imputing the baseline missing values

| Exposure | Non-adjusted | Adjust model |
| --- | --- | --- |
| Total difficulties | 0.98 (0.97, 0.99) <0.0001 | 0.98 (0.97, 0.99) <0.0001 |
| Prosocial behavior | 1.05 (1.04, 1.05) <0.0001 | 1.04 (1.03, 1.05) <0.0001 |

Note: OR (95%CI) P value
Non-adjusted model adjust for: None
Adjust model adjust for: parent respondent’s gender; parental age; child gender; child age; smoking status; alcohol intake status; education level; annual family income; employment status; marital status.
