# Supplementary material for: The association between parental involvement in developmental advance and mental health in Chinese preschoolers: a cross-sectional study
Source: Front Public Health. 2026 Jan 29;14:1677781. doi: 10.3389/fpubh.2026.1677781 (PMC12894225; doi:10.3389/fpubh.2026.1677781)
Supplement: Supplementary file 1 [file Data_Sheet_1.zip › Table 5 Absolute effect size .docx]

**Table 5 Absolute effect size**

| Outcome | Exposure | Exposure SD | Original OR | Original CI | OR per SD | Standardized Beta | Risk Low | Risk High | Risk Diff |
| --- | --- | --- | --- | --- | --- | --- | --- | --- | --- |
| Total Difficulties | PIDA | 3.76 | 0.98 | [0.97, 0.99] | 0.93 | -0.07 | 18.6 | 15.4 | -3.2 |
| Prosocial Behavior | PIDA | 3.76 | 1.04 | [1.03, 1.05] | 1.15 | 0.14 | 65 | 72.3 | 7.3 |
