# Supplementary figures and images for: The association between parental involvement in developmental advance and mental health in Chinese preschoolers: a cross-sectional study
Source: Front Public Health. 2026 Jan 29;14:1677781. doi: 10.3389/fpubh.2026.1677781 (PMC12894225; doi:10.3389/fpubh.2026.1677781)

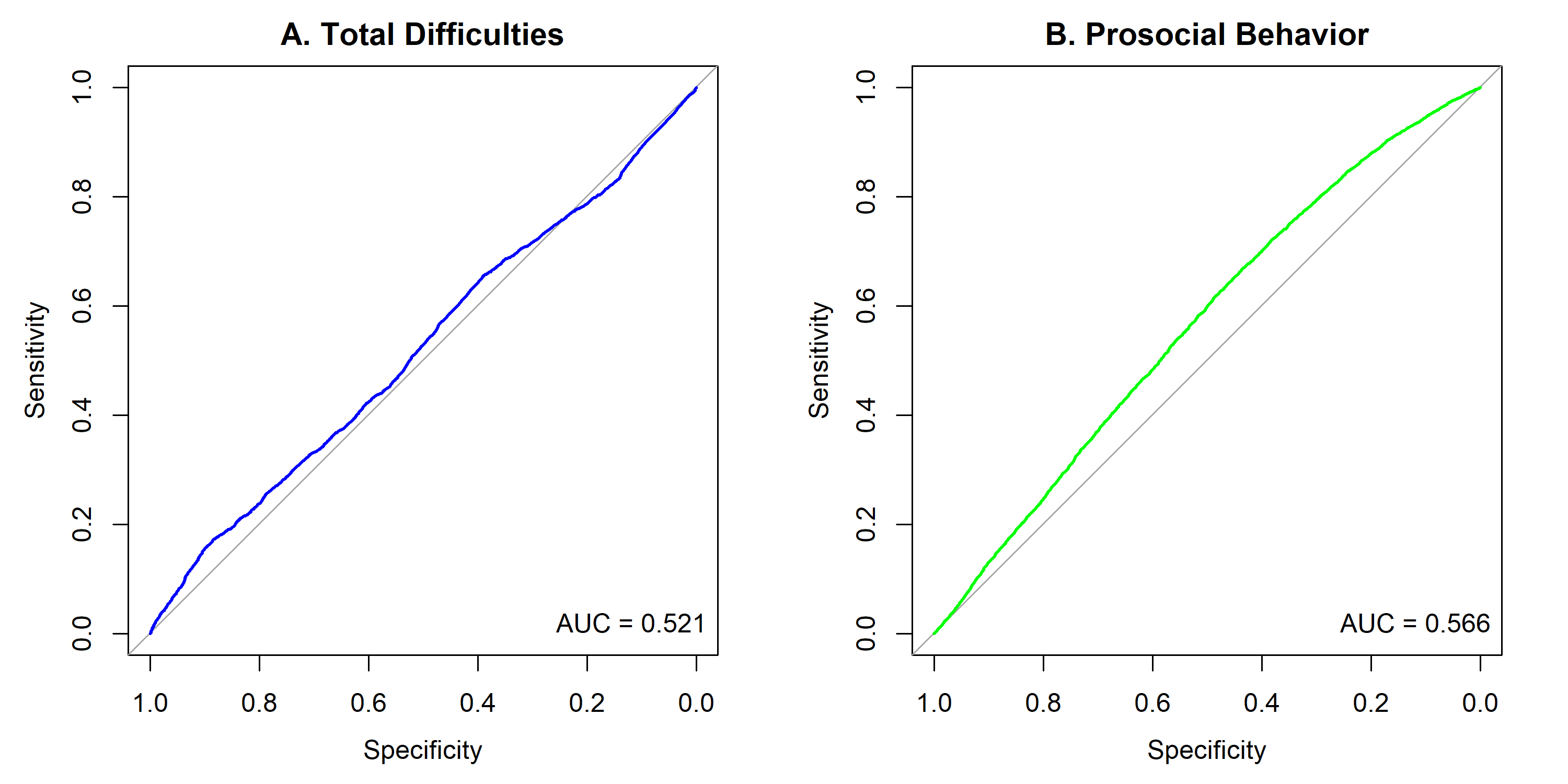

Supplement: Supplementary file 1 [file Data_Sheet_1.zip › Figure S1 ROC curves.png]
